# Nurses' Knowledge, Attitudes, and Practice in Relation to Pharmacovigilance and Adverse Drug Reaction Reporting: A Systematic Review

**DOI:** 10.1155/2021/6630404

**Published:** 2021-04-09

**Authors:** Tahmine Salehi, Naiemeh Seyedfatemi, Mohammad Saeed Mirzaee, Maryam Maleki, Abbas Mardani

**Affiliations:** ^1^Nursing Care Research Center, Department of Nursing Management, School of Nursing & Midwifery, Iran University of Medical Sciences, Tehran, Iran; ^2^Nursing Care Research Center, School of Nursing and Midwifery, Iran University of Medical Sciences, Tehran, Iran; ^3^School of Nursing & Midwifery, Tehran University of Medical Sciences, Tehran, Iran; ^4^Nursing Care Research Center, Department of Medical Surgical Nursing, School of Nursing and Midwifery, Iran University of Medical Sciences, Tehran, Iran

## Abstract

**Aim:**

To describe and synthesize aspects of knowledge, attitudes, and practice regarding pharmacovigilance and adverse drug reaction (ADR) reporting and to explore associated barriers from a nurse perspective.

**Methods:**

A systematic review was conducted. Electronic databases including MEDLINE, Embase, Scopus, and Web of Knowledge from January 2010 to October 2020 were searched. Original observational studies that were written in English and which focused on nurses' knowledge, attitudes, practice, and perceived barriers regarding pharmacovigilance and ADR reporting in various healthcare settings were included.

**Results:**

Twenty-three studies published in English from 2010 to 2020 were retrieved during the search process. Overall, in the knowledge domain, the median percentages of nurses who were aware of the definitions of ADRs were 74.1%, while only 26.3% were aware of the adverse drug reaction reporting form. In the attitude domain, 84.6% of nurses believed ADR reporting to be important for patient/medicine safety and 37.1% had a fear of legal liability following ADR reporting. Although 67.1% of nurses encountered ADRs during their professional life, only 21.2% had a history of ADR reporting. In addition, lack of knowledge/training (median: 47.1%) was identified as the most common barrier in ADR reporting from a nursing viewpoint.

**Conclusion:**

Despite positive nurse attitudes, knowledge and practice in relation to pharmacovigilance activities and ADR reporting did not occur regularly or often. Improving nurses' knowledge through in-service training and degree-level education and addressing the main barriers of ADR reporting may help to achieve an improved level of reporting.

## 1. Introduction

Adverse drug reaction (ADR) is defined by the World Health Organization (WHO) as “a response to a drug which is noxious and unintended, and which occurs at doses normally used in man for the prophylaxis, diagnosis, or therapy of disease, or for the modification of physiological function” [1]. ADRs are a developing and serious challenge for public health management due to the multiple comorbidities, polypharmacy, and arrival of new drugs on the market and are considered a major cause of patient morbidity and mortality [2–4]. It has been shown that ADRs account for 5%–10% of all hospital admissions [5, 6] and cause a 9% increase in the length of hospital stay and a 20% increase in the variety of care costs [7].

Pharmacovigilance (PV) refers to ADR reporting, defined by the WHO as “the science and activities relating to the detection, assessment, understanding, and prevention of adverse effects or any other possible drug-related problems” [1]. Although the range of PV activities consist of detection and reporting of medication errors, drug-to-drug interactions, misuse and/or abuse of medicines, lack of efficacy of medicines, and counterfeit and substandard medicines, ADRs remain the initial focus of PV activities [8]. National PV systems were developed by many countries after the thalidomide disaster in the 1960s [9]. These systems allow continuous monitoring of all drugs used in clinical settings and enable the creation of alerts for identifying new ADRs. However, their strength is entirely related to the actual rate reported by healthcare practitioners [10].

After approval of medicines, spontaneous ADR reporting is a foundation for the monitoring of a drug's benefit risk during the postmarketing phase [11]. This is necessary to identify unknown, unusual, and serious ADRs that may not have been discovered during the premarketing clinical trial phase or even during postmarketing supervision, to improve drug safety and understand the risks of drugs [8, 12]. Therefore, spontaneous ADR reporting can be used as a way to detect new, rare, or serious ADR events [13]. However, underreporting among healthcare providers is one of the major barriers to detect new and potential ADRs [14]. It is estimated that only 10% of ADRs are reported. Therefore, healthcare providers should be motivated and sensitized regarding ADR reporting [15].

A key to creating a more robust surveillance culture is to ensure that all healthcare professionals who administer drugs are aware of how to monitor and report any difficulties that patients may experience [8]. Also involving physicians and pharmacists, nurses should additionally play a proactive role in PV activities and ADR reporting [16]. Nurses have a unique position in the healthcare team to monitor a patient's response to medication as they administer most drugs in healthcare settings and they are often present when an ADR happens and are involved in taking appropriate action to ameliorate the problem accordingly [17, 18]. ADR reporting should be incorporated into the nurses' daily work schedule, and nurses therefore should have appropriate scientific training to enable them to be able to do this competently [19]. Increased engagement of nurses in ADR reporting can improve patient safety and reduce the costs of any ADR treatment complications [20]. However, previous literature has documented that a contribution from nurses is not optimal in the ADR reporting [16, 21, 22].

The rate of ADR reporting depends on many factors, such as national PV programs, regulations, and the knowledge and attitudes of healthcare professionals [23]. Evaluating knowledge, attitudes, and practice of healthcare providers toward PV and ADR reporting can help to devise strategies for improving reporting schemes to ensure patients safety. Hence, this systematic review was aimed at identifying the knowledge, attitudes, and practice toward PV and ADR reporting as reported by nurses and at considering associated barriers.

## 2. Methods

### 2.1. Protocol and Registration

This systematic review of international literature was aimed at considering all types of observational studies in relation to ADR [24, 25]. The Preferred Reporting Items for Systematic Reviews and Meta-Analyses (PRISMA (available here)) guidelines were used to report this systematic review [25]. In addition, this systematic review has been registered with PROSPERO under the code of CRD42020209145 which can be accessed at https://www.crd.york.ac.uk/prospero/display_record.php?ID=CRD42020209145.

### 2.2. Search Process and Eligibility Criteria

The research team discussed and agreed to the identification of appropriate search keywords based on the relevant literature. In addition, a pilot search in general and specialized databases was conducted to clarify identified relevant keywords. To retrieve studies about nurses' knowledge, attitudes, and practice toward PV and ADR reporting, the Boolean search method was used applying the following keywords: (nurs∗ AND (“drug-related side effects” and “adverse reactions” OR “adverse reaction” OR “fatal adverse drug reactions” OR “serious adverse drug reactions” OR “drug related side effects” OR “side effects” OR “adverse drug events” OR “suspected adverse drug reaction” OR “adverse drug reactions reporting” OR “reporting of adverse drug reactions” OR reporting OR “adverse event reporting” OR “adverse drug reaction reporting” OR pharmacovigilance OR “pharmacovigilance system” OR “drug monitoring program” OR “factors affecting reporting” OR underreporting OR “causes of underreporting” OR “drug event detection” OR “detecting adverse drug reactions”) AND (knowledge OR attitude OR practice OR behavior OR experience OR opinion OR perception OR awareness)). Accordingly, the online databases of Web of Knowledge, MEDLINE, Embase, and Scopus were searched to retrieve articles published in peer-reviewed journals from January 2010 to October 2020. To improve the search coverage, cross-references from the bibliographies of selected studies also were searched. Eligibility criteria for choosing relevant studies included all types of observational studies including survey-based, cross-sectional, and cohort studies which focused on the nurses' knowledge, attitudes, and practice toward PV and ADR reporting in various healthcare settings. All selected studies were published in peer-reviewed journals. Studies without objective relevance to nurses or concentration on the knowledge, attitudes, and practice toward PV and ADR reporting in the other healthcare professionals were excluded.

### 2.3. Study Selection

Three authors (AM, MSM, and MM) independently performed each step of the process of the systematic review as outlined in the search process. During the search process, the article titles, abstracts, and full texts were obtained and screened by the authors. Online discussions were held to share the results of searches completed and to decide on the subsequent steps of the systematic review. If there were disagreements, discussions were undertaken with a fourth author to reach a consensus about the inclusion of selected studies in the systematic review.

### 2.4. Quality Appraisal

The Enhancing the Quality and Transparency of Health Research (EQUATOR) tool was applied for quality appraisal-selected articles in terms of the research process and structure [26]. The appraisal tool Strengthening the Reporting of Observational Studies in Epidemiology (STROBE) was used for the cross-sectional study. In addition, Hawker et al.'s criteria toward the purpose of research, knowledge-based structure, quality of the methodology and research process, conclusion, and references were utilized in the appraising process [27]. In addition to attention to the scores obtained from the appraisal tool (Table 1), the authors' discussion helped to make appropriate decisions about the importance and the methodological quality of each study for the conclusive decision on the insertion or exclusion of studies and data analysis and synthesis.

### 2.5. Data Collection Process and Synthesis of Results

For data extraction, a table was developed by the authors. This table consisted of the authors' name, publication year, study location, design, sample size and setting of the study, and data related to nurses' knowledge, attitude, and practice toward PV and ADR reporting and barriers that prevent ADR reporting. A pilot test of four studies which was undertaken to ensure that this table enables gathering appropriate data from selected studies was effective.

To facilitate analysis and interpretation, the percent of positive and correct responses (responses were reversed when necessary) related to nurses' knowledge, attitude, and practice toward PV and ADR reporting was considered. Next, percentages of positive and correct responses were pooled and a median and interquartile range (IQR) was computed. A meta-analysis was not possible due to variation in the selected studies in terms of the samples, analytical strategies, and outcomes.

## 3. Results

### 3.1. Search Outcome and Selection of Studies

The results of the search process in the databases are presented in Table 2. A total of 5625 articles were retrieved during the search process applying the predetermined keywords. Finally, twenty-three studies were selected for data analysis and synthesis after removing irrelevant and duplicate titles and conducting abstract and full-text reading phase. During the full-text appraisal phase, the methodological quality of the selected articles was evaluated. No study was ruled out because it was judged to be of an unacceptable quality in terms of theoretical and conceptual framework and research design.

**Figure 1:**
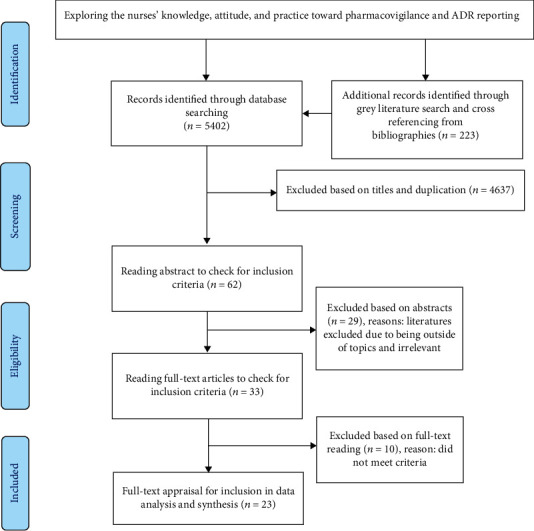
The Preferred Reporting Items for Systematic Reviews and Meta-Analyses (PRISMA (available here)).

The flow diagram of the study based on the Preferred Reporting Items for Systematic Reviews and Meta-Analyses (PRISMA (available here)) is presented in Figure 1.

### 3.2. General Characteristics of the Selected Studies

General characteristics of the selected studies (*n* = 23) have been shown in Table 1. All studies were published in English from 2010 to 2020. Four studies were from India [28–31], three from South Africa [32–34], three from Turkey [35–37], two from Saudi Arabia [38, 39], two from Jordan [40, 41], two from Pakistan [42, 43], two from Nepal [44, 45], one from Bhutan [46], one from Sweden [47], one from Iran [48], one from the United Arab Emirates [49], and one from Ethiopia [50].

All studies used a cross-sectional questionnaire-based study design except one study which applied a retrospective observational, prospective cross-sectional design [31]. All studies except three [36, 41, 47] were conducted mainly in the hospitals. The majority of the included studies involved multihealthcare professions as participants, and only a few studies involved nurses as participants [30, 37, 47–49]. The total number of nurse participants in the selected studies was 3672. The tools used in most of the included studies were developed commonly to evaluate knowledge, attitude, and practice altogether toward pharmacovigilance and ADR reporting.

### 3.3. Main Findings

The main findings of this review have been presented separately for nurses' knowledge, attitude, practice, and perceived barriers concerning PV activities and ADR reporting.

#### 3.3.1. Nurses' Knowledge toward PV Activities and ADR Reporting

For the evaluation of nurses' knowledge regarding PV and ADRs, six items were developed as follows: PV definition, ADR definition, knowledge of ADR reporting, awareness of ADR reporting form, awareness of the national PV system, and receiving training about PV and ADR reporting. In the present review, four of the included studies had no data for the items developed in the knowledge domain [30, 31, 41, 47].

Nurses who had awareness of ADR and PV definition were 34.0% (median percentages) (IQR: 25.3-49.5) and 74.1% (IQR: 55.2-81.2), respectively. In addition, 50% (IQR: 44.2-82.6) of the nurses had knowledge of ADR reporting, and surprisingly only 26.3% (IQR: 16.6-54.6) of them had awareness of the ADR reporting form. It was also found that only 31.6% (IQR: 15.5-50.2) of nurses were aware of the national pharmacovigilance system and 38.7% (IQR: 4.0-73.2) of them had training about PV and ADR reporting (Table 3).

#### 3.3.2. Nurses' Attitude toward PV Activities and ADR Reporting

Nurses' attitudes regarding PV and ADR reporting were assessed through six items including ADR reporting being important for patient/medicine safety, ADR reporting being a professional commitment, ADR reporting being necessary, ADR reporting being mandatory or voluntary, and fear of legal liability following ADR reporting. Of the 23 included studies in this review, seven studies did not supply any data for the items developed in the attitude domain [31, 36, 38, 41, 42, 46, 47].

According to the results, 84.6% (IQR: 71.1-89.7) of the nurses acknowledged that ADR reporting is important for patient/medicine safety. Also, 71.4% (IQR: 60.4-77.9) of them believed that ADR reporting is a professional commitment and 66.7% (IQR: 49.7-75.0) of them were thinking that ADR reporting is necessary. Similarly, 76.5% of the nurses believed that ADR reporting should be mandatory and 72.2% (IQR: 39.3-81.6) believed that ADR reporting should be voluntary. Furthermore, the nurses who had fear of legal liability following ADR reporting were 37.1% (IQR: 35.8-43.8) (Table 3).

#### 3.3.3. The Practice of ADR Reporting among Nurses

Three items including advising patients on possible adverse reactions, history of encountering an ADR episode with a patient, and history of ADR reporting were applied to assess nurse practice of ADR reporting. Six of the included studies in the review did not provide any information for the items applied in the practice domain [28, 33, 41, 44, 46, 48].

It was found that 53.6% (IQR: 40.5-71.0) of the nurses had experiences of advising patients on possible ADR. In addition, although 67.1% (IQR: 43.4-75.5) of the nurses had a history of encountering a patient with ADR, only 21.2% (IQR: 8.6-41.7) of them had the experience of ADR reporting (Table 3).

#### 3.3.4. Barriers toward PV and ADR Reporting among Nurses

Of 23 included studies in the review, twelve studies provided data about barriers to ADR reporting experienced by nurses [28, 30, 31, 35, 36, 40–44, 47, 49]. Lack of knowledge/training (median: 47.1%) was the most common barrier in ADR reporting from the nurses' opinion which was cited across all twelve studies. Well-known ADRs (43.9%), lack of promotion and reminders by the authorities (43.5%), lack of information provided by patients (42%), lack of access to ADR forms (38.5%), confidentiality/legal problems (34.6%), lack of time (31.5%), uncertainty in diagnosis (29.8%), lack of importance of ADR reporting (25.2%), lack of motivation/feedback (17.9%), and believing that ADR reporting was not the responsibility of the nurse (15.9%) were in the next set of ranks identified as barriers to ADR reporting (Table 4).

## 4. Discussion

ADRs are one of the most crucial health problems worldwide. Among diverse factors that influence PV activities and ADR reporting, knowledge, attitude, and practice of healthcare professionals have a considerable role [51]. This systematic review synthesized nurses' knowledge, attitudes, and practice and associated barriers regarding PV and ADR reporting.

Findings from our review indicate that knowledge held by nurses is not at the desired level in PV definition (34%), knowledge of ADR reporting (50%), awareness of ADR reporting form (26.3%), and awareness of the national PV system (31.6%). It was shown that knowledge had a strong influence on ADR reporting and lack of knowledge is one of the major obstacles for ADR reporting [52]. Similarly, a systematic review in India reported that 55.6% of the health professionals were not aware of the national pharmacovigilance program [53]. In another systematic review in Ethiopia, 45.9% of health professionals were aware of the national ADR reporting system and their knowledge of ADR was determined (41.5%) [54]. Therefore, it seems that healthcare authorities should increase nurses' knowledge about the PV system and also facilitate nurses' awareness of ADR reporting form by adopting appropriate strategies.

According to our review findings, nurse attitudes were at a higher level than their knowledge and practice toward PV and ADR reporting. Although our review findings highlighted that 71.4% of nurses acknowledged that ADR reporting is a professional commitment, nurses' limited awareness about their key professional role in pharmacovigilance activities is one of the main factors influencing PV activities and ADR reporting [55]. Furthermore, the review findings showed that more than two-thirds of nurses believed that ADR reporting is necessary and important for patient/medicine safety.

In addition, nurses almost equally believed that ADR reporting should be mandatory or voluntary. It is generally known that spontaneous reporting programs, where reports are submitted voluntarily, are associated with fairly low levels of ADR reporting [56]. Therefore, a high rate of underreporting of ADRs can postpone signal detection and thus endanger patients' safety [57]. Findings of the study by Rehan et al. suggest that more than half of the nurses and resident doctors believe that PV activities including ADR reporting should be a mandatory practice to ensure and improve patient safety [58]. Another study found that lack of mandatory regulation on ADR reporting affected medical staff confidence to undertake ADR reporting when experienced clinically [59]. However, subjectivity in ADR identification makes it hard to perform mandatory reporting by healthcare providers. It seems that giving obvious guidance to health professionals that highlights the benefits of ADR reporting in increasing medication safety knowledge could enhance the feasibility and effectiveness of mandatory reporting methods [60].

Our review findings indicate that although 67.1% of nurses encountered patients with ADR during their clinical practice, only a small percentage (21.2%) of them had the experience of ADR reporting. In several studies, nurses acknowledged that they were not adequately prepared to be capable to report ADRs [16, 61]. Similar to our findings, a systematic review by Bhagavathula et al. showed that 74.5% of Indian healthcare professionals including nurses never reported any ADRs [53]. In addition, the poor practice of ADR reporting among doctors was reported in another systematic review [62]. This review also identified that 53.6% of nurses told patients about possible ADR. Previous literature suggested that the engagement of patients in medication monitoring and patient safety activities has an important impact in increasing patients' safety during hospitalization [63]. Therefore, by increasing patients' awareness of ADR and their engagement in medication monitoring, ADR identification and reporting can be increased.

Underreporting of ADRs is one of the most important problems related to PV programs. As shown by nurse viewpoints in the findings of this review, lack of knowledge/training was the most significant barrier that influenced ADR reporting. Consistent with our finding, a systematic review by Varallo et al. reported that lack of knowledge in completing the ADR form is one of the main causes of underreporting among nurses [64]. Another systematic review suggested that nurses' belief about insufficient pharmacology knowledge to identify an ADR is an important reason for underreporting [55]. As shown in this study, only 38.7% of nurses had a history of training about PV and ADR reporting. The available literature acknowledges that higher education and provision of training for nurses would be associated with a greater engagement in ADR identification and reporting [65, 66]. In addition, previous studies confirmed that completing nurses' training related to PV is pivotal to optimizing their roles in PV practices [67, 68].

Nurses can acquire pharmacological knowledge from theoretical and practical training courses during the nursing educational program, in-service training, and clinical experience. Therefore, provision of degree-level education and in-service training for nursing staff with appropriate educational strategies such as high-fidelity simulation, problem-based learning, role modeling, reflection and discussion, interprofessional education, and case study learning may help the development of competencies and skills associated with the PV and reporting of ADRs [69–71].

Evidence from various studies suggested that lack of time [72] and lack of awareness of where and how to report the suspected ADRs [73] are already well known [56]. In addition, lack of recognition of the importance of ADR reporting [74], uncertainty about the ARD diagnosis [75, 76], fear of legal consequences [77], difficulty in filling out the ADR form [78], and lack of access to ADR forms [78] were some of the additional factors for underreporting of ADRs by health professionals which is consistent with our study findings. Improvement and modification of these features in healthcare settings could increase the rates of ADR reporting.

### 4.1. Strengths and Limitations

Our study is the first that evaluates the knowledge, attitudes, and practice of nurses toward PV activities and ADR reporting internationally by including 23 studies from across the world. However, our review is not without limitations. We excluded studies in which nurses were participants along with other healthcare professions if a separate analysis for nurses' knowledge, attitudes, and practice was not included. In addition, studies were limited to the English language. However, using multidimensional keywords and in international databases during the search process, a comprehensive view of the present international knowledge about the nurses' knowledge, attitudes, and practice toward PV activities and ADR reporting was provided. Furthermore, the bias in the process of the review was decreased as much as possible using close cooperation, critical considerations, and conversation between the authors.

## 5. Conclusion

This systematic review focused on nurses' knowledge, attitudes, and practice toward pharmacovigilance and ADR reporting and the associated barriers. Although nurses have a positive attitude toward PV and ADR reporting, their knowledge and practice in pharmacovigilance activities and ADR reporting were not at a suitable level of competence. In addition, lack of knowledge/training was the most significant barrier that influenced ADR reporting. Considering the critical role of nurses in PV activities and ADR reporting, sufficient attention should be paid to in-service training and degree-level education for nurses to ensure that this competence can be addressed. Also, facilitating access to the ADR reporting form, applying online submission of ADR reports, simplifying the ADR reporting process, and implementing electronic reporting and providing motivation and feedback can increase ADR reporting performance. However, it is recommended that future studies applying qualitative and quantitative research designs should investigate how nurses can be more actively engaged in ADR reporting.

## Figures and Tables

**Table 1:** General characteristics of the included studies.

Authors, year	Country	Study design/full-text appraisal score	Study setting	Sampling method	Sample size
Abdel-Latif and Abdel-Wahab [38]	Saudi Arabia	A cross-sectional questionnaire-based study/22 out of 32	9 hospitals	Random sampling	158
Abu Hammour et al. [40]	Jordan	A cross-sectional questionnaire-based study/24 out of 32	One hospital	Convenience sampling	214
Ahmed et al. [42]	Pakistan	A cross-sectional questionnaire-based study/17 out of 32	One hospital	Unclear	25
Al Rabayah et al. [41]	Jordan	A cross-sectional questionnaire-based study/17 out of 32	One cancer center	Unclear	154
AlShammari and Almoslem [39]	Saudi Arabia	A cross-sectional questionnaire-based study/21 out of 32	Nine hospitals	Random sampling	110
Bepari et al. [28]	India	A cross-sectional questionnaire-based study/18 out of 32	One hospital	Convenience sampling	64
Bogolubova et al. [32]	South Africa	A cross-sectional questionnaire-based study/24 out of 32	Six hospitals	Purposive sampling	6183
Danekhu et al. [44]	Nepal	A descriptive, cross-sectional questionnaire-based study/26 out of 32	One hospital	Stratified random sampling	126
Dorji et al. [46]	Bhutan	A cross-sectional questionnaire-based study/21 out of 32	Four hospitals	Census sampling	257
Ekman et al. [47]	Sweden	A cross-sectional questionnaire-based study/25 out of 32	Nurses who are members of the Swedish Association of Health Professionals	Random sampling	453
Ergün et al. [35]	Turkey	A cross-sectional questionnaire-based study/16 out of 32	One hospital	Unclear	321
Ganesan et al. [29]	India	A cross-sectional questionnaire-based survey/18 out of 32	One hospital	Unclear	171
Gordhon and Padayachee [33]	South Africa	A cross-sectional questionnaire-based study/23 out of 32	One hospital	Stratified sampling	230
Güner and Ekmekci [36]	Turkey	A cross-sectional questionnaire-based study/20 out of 32	Online survey	Convenience sampling	67
Hanafi et al. [48]	Iran	A cross-sectional questionnaire-based study/22 out of 32	One hospital	Census sampling	224
John et al. [49]	United Arab Emirates	A cross-sectional questionnaire-based study/25 out of 32	One hospital and one research center	Census sampling	91
Rajalakshmi et al. [30]	India	A cross-sectional questionnaire-based study/15 out of 32	One hospital	Unclear	101
Santosh et al. [45]	Nepal	A cross-sectional questionnaire-based study/18 out of 32	Four hospitals	Unclear	135
Shamim et al. [43]	Pakistan	A cross-sectional questionnaire-based study/21 out of 32	Five hospitals and an orthopedics and medical institute	Unclear	69
Shanko and Abdela [50]	Ethiopia	A cross-sectional questionnaire-based study/26 out of 32	One hospital	Purposive sampling	230
Tandon et al. [31]	India	A retrospective observational, prospective cross-sectional study/18 out of 32	One hospital	Quota sampling	100
Terblanche et al. [34]	South Africa	A cross-sectional questionnaire-based study/21 out of 32	One hospital	Convenience sampling	77
Vural et al. [37]	Turkey	A cross-sectional questionnaire-based study/20 out of 32	One hospital	Census sampling	112

**Table 2:** The search strategy and results of different phases of the study.

Databases from 2010 to 2020	Total in each database	Title selection	Abstract selection	Full-text appraisal
MEDLINE	1702	12	10	7
Scopus	1529	6	3	1
Embase	794	31	14	11
Web of Science	1377	8	5	3
Manual search/backtracking references	223	5	1	1
Total of databases	5625	62	33	23

**Table 3:** Nurses' knowledge, attitude, and practice toward pharmacovigilance and ADR reporting.

Author	Domains														
	Knowledge (% yes)						Attitude (% yes)						Practice (% yes)		
	PV definition	ADR definition	Knowledge of ADR reporting	Awareness of ADR reporting form	Awareness of the national PV system	Receiving training about PV and ADR reporting	ADR reporting important for patient/medicine safety	ADR reporting is a professional commitment	ADR reporting is necessary	ADR reporting should be mandatory	ADR reporting should be voluntary	Fear of legal liability following ADR reporting	Advising patients on possible ADR	History of encountering a patient with ADR	History of ADR reporting
Abdel-Latif and Abdel-Wahab [38]			99.3		27.2								73.4		
Abu Hammour et al. [40]	36.0	79.0	50.0				89.7	74.8				34.6			
Ahmed et al. [42]			60.0	28.0										72.0	72.0
AlShammari and Almoslem [39]					16.0					70.0					21.0
Bepari et al. [28]	26.6					89.1		4.7	45.3						
Bogolubova et al. [32]					23.6	68.0	81.4		76.6						21.5
Danekhu et al. [44]	46.0	54.8		6.3	14.3		59.5								
Dorji et al. [46]		55.6	85.2												
Ekman et al. [47]															14.0
Ergün et al. [35]	60.0				36.0		75.0				91.0			41.0	21.0
Ganesan et al. [29]				54.0	36.0	5.0		67.0							25.0
Gordhon and Padayachee [33]			46.4					88.9							
Güner and Ekmekci [36]						22.4							43.3		
Hanafi et al. [48]	32.1		34.8	20.1								37.1			
John et al. [49]		83.5			49.5		87.9							82.4	8.8
Rajalakshmi et al. [30]							90.0					50.5	39.6	73.2	28.7
Santosh et al. [45]			80.0		57.8			77.8	63.0						36.3
Shamim et al. [45]			42.0	24.6	10.1	55.1		68.1						62.3	66.7
Shanko and Abdela [50]	21.7			56.5	52.6			58.2			72.2		63.9	44.3	58.2
Tandon et al. [31]															4.1
Terblanche et al. [34]						1.3		78.0		83.1	6.5				8.0
Vural et al. [37]		74.1	50.0						70.5						8.0
Median (IQR)	34.0 (25.3-49.5)	74.1 (55.2-81.2)	50.0 (44.23-82.60)	26.3 (16.6-54.6)	31.6 (15.5-50.2)	38.7 (4.0-73.2)	84.6 (71.1-89.7)	71.4 (60.4-77.9)	66.7 (49.7-75.0)	76.5	72.2 (39.3-81.6)	37.1 (35.8-43.8)	53.6 (40.5-71.0)	67.1 (43.4-75.5)	21.2 (8.6-41.7)

ADR: adverse drug reaction; IQR: interquartile range; PV: pharmacovigilance.

**Table 4:** Barriers toward ADR reporting by the nurses.

Author, year	Barriers to ADR reporting (%)											
	Lack of access to ADR forms	Lack of time	Lack of knowledge/training	Lack of motivation/feedback	Confidentiality/legal problem	Uncertainty in diagnosis	Lack of information provided by patients	Lack of promotion by the authorities	Difficulty in filling the form	Lack of importance	Not my responsibility	Well known ADRs
Abu Hammour et al. [40]		57.0	53.0		34.6		68.2	48.1		25.2		
Ahmed et al. [42]			36.0			52.0			36.0	20.0		
Al Rabayah et al. [41]	7.0	27.0	33.0	19.0		2.0				6.0	17.0	
Bepari et al. [28]			12.5		9.4	23.4				54.7		
Danekhu et al. [44]		0.8	44.4				15.9	38.9				
Ekman et al. [47]	39.0	30.0	51.0			37.0						56.0
Ergün et al. [35]	38.0	40.0	52.0		53.0	27.0			36.0	36.0		
Güner and Ekmekci [36]		3.0	10.5			14.9					14.9	
John et al. [49]		33.0	45.1			49.5						31.9
Rajalakshmi et al. [30]			50.4	16.8		32.6				18.8		
Shamim et al. [43]	59.4	55.0	49.2			26.1						
Tandon et al. [31]			91.0			82.0			88.0	91.0		
Median (IQR)	38.5 (14.7-50.2)	31.5 (9.0-51.2)	47.1 (33.7-51.7)	17.9	34.6 (22.0-43.8)	29.8 (21.2-50.1)	42.0	43.5	36.0	25.2 (18.8-57.7)	15.9	43.9

ADRs: adverse drug reactions.

## Supplementary Materials

Supplementary Materials The Preferred Reporting Items for Systematic Reviews and Meta-Analyses (PRISMA) checklist.
